# Development of the sleep-wake switch in rats during the P2-P21 early infancy period

**DOI:** 10.3389/fnetp.2023.1340722

**Published:** 2024-01-04

**Authors:** Mainak Patel, Badal Joshi

**Affiliations:** ^1^ Department of Mathematics, William & Mary, Williamsburg, VA, United States; ^2^ Department of Mathematics, California State University San Marcos, San Marcos, CA, United States

**Keywords:** locus coeruleus, sleep wake, infant development, reciprocal inhibition, power law

## Abstract

In early infancy, rats randomly alternate between the sleeping and waking states–from postnatal day 2–10 (P2-P10), sleep and wake bouts are both exponentially distributed with increasing means, while from P10-P21 sleep and wake bout means continue to increase, though there is a striking qualitative shift in the distribution of wake bouts from exponential to power law. The behavioral states of sleep and wakefulness correspond to the activity of sleep-active and wake-active neuronal brainstem populations, with reciprocal inhibition between the two ensuring that only one population is active at a time. The locus coeruleus (LC) forms a third component of this circuit that rises in prominence during the P10-P21 period, as experimental evidence shows that an as-of-yet undeciphered interaction of the LC with sleep-active and wake-active populations is responsible for the transformation of the wake bout distribution from exponential to power law. Interestingly, the LC undergoes remarkable physiological changes during the P10-P21 period–gap junctions within the LC are pruned and network-wide oscillatory synchrony declines and vanishes. In this work, we discuss a series of models of sleep-active, wake-active, and the LC populations, and we use these models to postulate the nature of the interaction between these three populations and how these interactions explain empirical observations of sleep and wake bout dynamics. We hypothesize a circuit in which there is reciprocal excitation between the LC and wake-active population with inhibition from the sleep-active population to the LC that suppresses the LC during sleep bouts. During the P2-P10 period, we argue that a noise-based switching mechanism between the sleep-active and wake-active populations provides a simple and natural way to account for exponential bout distributions, and that the locked oscillatory state of the LC prevents it from impacting bout distributions. From P10-P21, we use our models to postulate that, as the LC gradually shifts from a state of synchronized oscillations to a state of continuous firing, reciprocal excitation between the LC and the wake-active population is able to gradually transform the wake bout distribution from exponential to power law.

## 1 Introduction

The sleep-wake system remains an area of active investigation, with models often focusing on cortical phenomena or the dynamics of the brainstem sleep-wake circuit as REM/NREM sleep emerges and hypothalamic and circadian influences on sleep-wake switching become more prominent (Booth and Behn, 2014). In adult rats, which exhibit resting periods during both the light and dark phases of the 24 h cycle, although with a greater proportion of time spent in the wake state during the dark phase (Cui et al., 2019), sleep-wake circuit physiology has received considerable experimental attention. However, the physiology of the sleep-wake circuit very early during infancy (corresponding to postnatal 2 (P2) to postnatal day 21 (P21) in rats) remains largely to be elucidated, with few models addressing the development or dynamics of the system during this early period. During the early postnatal period, however, the brainstem sleep-wake system is relatively simple–brainstem populations of sleep-active neurons have yet to differentiate into REM-ON and REM-OFF subpopulations (i.e., the behavioral state of sleep has yet to subdivide into alternating REM and non-REM substates), the suprachiasmatic nucleus of the hypothalamus (which entrains its activity to the external light/dark cycle) has yet to establish a significant impact on the brainstem (hence a lack of an imposition of circadian rhythms on the brainstem sleep-wake circuit), the wake-promoting hypocretin-producing neurons of the lateral hypothalamus have yet to develop significant connections with or exert substantial influence over brainstem sleep-active and wake-active populations, and the wake-promoting noradrenergic neurons of the locus coeruleus have yet to impact the dynamics of sleep-wake switching (until the latter part of the early postnatal period) (Blumberg et al., 2014; Frank, 2020). Thus, understanding the development and dynamics of the brainstem sleep-wake system during this early period, in the absence of such complicating influences, may provide a valuable framework for gaining insight into its functioning later in life as these influences become more prominent.

During the P2-P21 period, infant rats rapidly cycle between the states of sleep and wakefulness, with a random amount of time spent in each sleep or wake bout; indeed, the amount of time spent in a sleep or wake bout is independent of the length of prior bouts. From P2-P10, both sleep and wake bouts are exponentially distributed, with mean bout times increasing during this period while the exponential nature of the bout time distributions persists–mean sleep bout length increases from ∼15 to ∼35 s, while mean wake bout length increases from ∼5 to ∼10 s (Kleitman and Engelmann, 1953; Lo et al., 2002; Halász et al., 2004; Karlsson et al., 2004; Lo et al., 2004; Blumberg et al., 2005; Karlsson et al., 2005; Gall et al., 2009). During the P10-P21 period, however, a striking shift occurs in the distribution of sleep and wake bout lengths–while sleep bouts continue to remain exponentially distributed (with the mean increasing to ∼70 s), the wake bout distribution shifts gradually from exponential to a more heavy-tailed, power law-like distribution as its mean increases to ∼25 s (Kleitman and Engelmann, 1953; Karlsson et al., 2004; Blumberg et al., 2005; Karlsson et al., 2005; Gall et al., 2009).

While a detailed description of the brainstem physiology underpinning these early developmental behavioral observations remains elusive, some clues can be gleaned from experimental investigations. Behavioral sleep and wake bouts are mirrored by the activity of mutually inhibitory brainstem populations termed sleep-active cells and wake-active cells–during a wake bout, sleep-active cells are suppressed while wake-active neurons spike, with the opposite activity profile seen during a sleep bout. Wake-active populations reside in a thalamic branch (e.g., laterodorsal tegmentum, pedunculopontine tegmentum) and a hypothalamic branch (e.g., dorsal raphe nuclei, tuberomamillary nucleus), while sleep-active populations include the ventrolateral preoptic area, medullary inhibitory area, nucleus pontis oralis, and subcoeruleus (Blumberg et al., 2005; Karlsson et al., 2005; Schwartz and Roth, 2008). A key player during early development of the sleep-wake system is the locus coeruleus (LC), a small population of noradrenergic neurons which is known to diffusely innervate numerous brainstem populations comprising the early sleep-wake system (Gall et al., 2009). Experimental evidence suggests that while the influence of the LC on the brainstem sleep-wake circuit may be minimal during the P2-P10 period, it is critical during the P10-P21 period, and that the interaction of the LC with sleep-active and wake-active populations from P10-P21 plays a strong causal role in the observed transformation of the wake bout distribution from exponential to power law during this epoch. Indeed, if the LC is lesioned prior to P10, then the development of sleep bouts during P10-P21 is unaffected, but wake bouts, though continuing to exhibit an increasing mean bout length, fail to develop a power law distribution and remain exponential (Aston-Jones and Bloom, 1981; Saper et al., 2001; Gall et al., 2009; Takahashi et al., 2010; Berridge et al., 2012). Unfortunately, the nature of the interaction between the LC and sleep-active and wake-active populations, particularly during the pivotal P10-P21 period, remains empirically unknown.

Interestingly, and in temporal concordance with the waxing influence of the LC on the sleep-wake circuit during the P10-P21 period, there occurs a substantial shift in the internal physiology and dynamics of the LC network during this period. During the P2-P10 period, LC cells are extensively interconnected via slow dendrodendritic gap junctions, and the LC exhibits network-wide subthresold membrane potential synchrony and globally coordinated, large-amplitude oscillations, with a frequency of ∼0.3 Hz and up to 15 mV in amplitude. This globally oscillatory regime, thought to be due to both gap junction coupling (causing synchronous spiking) as well as intra-network synaptic inhibition (leading to oscillations), persists throughout the P2-P10 epoch. During the P10-P21 period, however, a progressive pruning of gap junctions between LC cells occurs, along with desynchronization of the LC network–as gap junctions are pruned from P10-P21, the frequency of the global LC oscillation increases up to ∼3 Hz while the amplitude dampens, and at P21 and beyond gap junction connectivity is eliminated and global synchrony is rarely seen under normal conditions (Coyle and Molliver, 1977; Groves and Wilson, 1980; Williams and Marshall, 1987; Christie et al., 1989; Christie and Jelinek, 1993; Travagli et al., 1995; Ishimatsu and Williams, 1996; Christie, 1997). The drastic changes in LC physiology that occur precisely during the transition of the wake bout distribution from exponential to power law are suggestive of a link between the two, though the nature of such a link remains empirically unresolved.

In this paper, we review a series of models that we have developed of the rat sleep-wake system through the early P2-P21 period (Patel and Joshi, 2014; Patel, 2015; Patel and Joshi, 2015; Patel and Rangan, 2017). Schematically, the model employs three interacting populations (a sleep-active population, a wake-active population, the LC), and we use our modeling work to present an overarching, experimentally testable theory of the development of these three populations during the P2 to P21 epoch. We begin by presenting our theory of the dynamics of the two-population system consisting of a sleep-active and a wake-active population, and describe how our model parsimoniously explains the available behavioral observations on sleep and wake bout development during the P2-P10 period. We then discuss our biophysical model of the LC, positing how gap junction pruning during the P10-P21 period leads to the physiological changes in LC dynamics described above. Finally, we discuss the full three-population model, and present our hypothesis pertaining to the mechanisms by which changing internal LC dynamics during P10-P21 fundamentally alter its interactions with sleep-active and wake-active populations and lead to the transition of the wake bout distribution from exponential to power law.

## 2 Sleep-wake switching during the P2-P10 period

The exponential distribution of sleep and wake bouts during the P2-P10 epoch has interesting mathematical implications. The exponential distribution is characterized by memorylessness–a distribution is exponential if and only if it is memoryless. Memorylessness, in the context of sleep and wake bouts, implies that, in the midst of a bout, the remaining length of the bout exhibits no dependence on the current duration of the bout; in other words, the system has no means by which to keep track of the ongoing duration of a bout. Another way to phrase this is to state that the hazard rate–the instantaneous probability of a bout switch–is constant throughout the duration of a sleep or wake bout. From a dynamical systems perspective, sleep and wakefulness represent two deterministically stable states within this bistable system, with the sleep state characterized by sleep-active neurons firing and wake-active neurons suppressed, and wakefulness characterized by the reciprocal activity pattern. In a deterministic setting, the system will settle into one state or the other permanently–in the presence of noise, however, the system will randomly switch back and forth between the two stable states (van Kampen, 2007; Gardiner, 2009).

**FIGURE 1:**
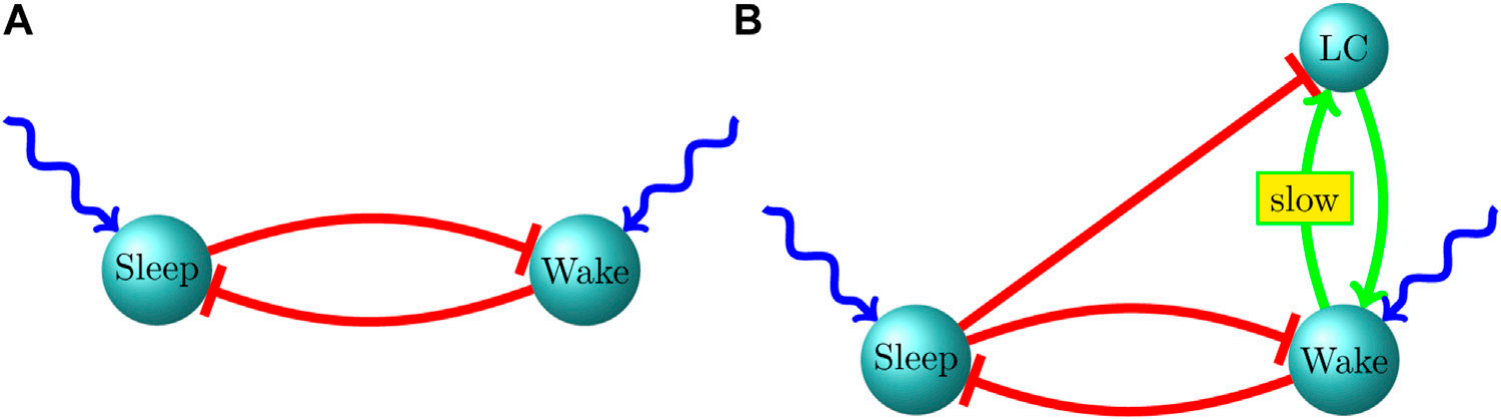
Our proposed circuit for the infant sleep-wake system. **(A)** P2-P9, **(B)** P10-P21.

Thus, in our work (Patel and Joshi, 2014; Patel, 2015) we model sleep-active and wake-active populations within the P2-P10 period simply as two biophysically simulated popuations of neurons that strongly inhibit each other, with each population receiving a noisy excitatory current (with constant mean) from outside the two-population system (Figure 1A). In this scenario, there are only three vehicles for the creation of memory within the system: 1) cross-population synaptic inhibition; 2) the noisy excitatory current to the two populations; 3) intrapopulation synapses. We find in this model that on the time scale of bouts (several to a few tens of seconds), none of these mechanisms is likely to create memory within the system, leading to sleep and wake bouts that appear exponentially distributed. The inhibitory current to the suppressed population rapidly (relative to typical bout durations) equilibrates after the onset of a bout, exhibiting a fixed mean level quickly after bout onset and hence lacking the ability to track the ongoing duration of the bout. The noisy excitatory current, since its mean is fixed and fluctuations are fast relative to the time scale of typical bouts, is also incapable of tracking ongoing bout duration, while an argument similar to that for cross-population inhibition holds for intrapopulation synapses as well–intrapopulation activity level in the active population rapidly (relative to the time scale of typical bouts) approaches a fixed mean value after bout onset, and is therefore unable to create memory and break the exponential nature of bout distributions.

One advantage of this model is its parsimonious nature. Including an element such as a homeostatic sleep drive that builds gradually during a wake bout, as is often incorporated in models of post-infancy sleep-wake switching (Tamakawa et al., 2006; Phillips and Robinson, 2007; Rempe et al., 2010; Kumar et al., 2012; Dunmyre et al., 2014), would introduce memory into the system (the building sleep drive would provide a measure of the current duration of a wake bout). Even in models in which stochastic elements are included (Behn et al., 2007; Diniz Behn and Booth, 2010), the switching mechanism fundamentally remains the deterministic sleep drive. See (Booth and Behn, 2014) for a review. In order to retain exponential bout durations, there would need to be a finely balanced interplay of deterministic and stochastic elements such that the system continues to appear memoryless (i.e., the hazard rate remains constant during a bout). Furthermore, these models tend to address a more mature sleep-wake system, in which external influences, such as from the cortex or hypothalamus or entraining by circadian/ultradian rhythms, tend to influence the dynamics of sleep-active and wake-active populations. During the early P2-P10 period, these external influences tend to be absent, and sleep and wake bouts tend to be short, random, and exponentially distributed; we therefore suggest that the exponential bout durations (and the associated constant hazard rates during sleep or wake bouts) seen during the P2-P10 period are more likely to arise simply and naturally as a consquence of stochastic switching within a bistable system (i.e., a system simply receiving noisy, constant-mean external excitation).

The model also provides an explanation of the increase in mean sleep and wake bout times during the P2-P10 period while the exponential nature of bout distributions persists. Either increasing the magnitude (or temporal duration) of cross-population synaptic inhibition or strengthening the noisy excitatory current received by the two populations can accomplsh this; the former, however, provides distinct advantages that the latter does not. Within the model, strengthening inhibition from the sleep-active to the wake-active population increases the mean sleep bout time while leaving the mean wake bout time unaffected (and *vice versa*). We term this phenomenon *independent control* of sleep and wake bout times (Patel and Joshi, 2014; Patel, 2015), and it occurs because if inhibition from population *A* to population *B* is strengthened, then during a bout of population *A* there is a greater mean level of inhibition delivered to population *B* (tending to prolong bouts of population *A*), while during a bout of population *B* bout dynamics are unaffected (since population *A* is quiescent, and hence inhibition from population *A* to population *B* is absent and can have no impact on the duration of population *B*’s bout). Strengthening the noisy excitatory drive to population *A*, on the other hand, affects the duration of the bouts of *both* populations–during a bout of population *A*, the increased excitation to population *A* elevates its firing rate and causes a greater mean level of inhibition to be delivered to population *B* (prolonging bouts of population *A*), while during a bout of population *B*, the strengthened excitation to population *A* increases the hazard rate (tending to shorten bouts of population *B*).

Thus, we find that modifying cross-population synaptic inhibition provides a mechanism for changing the mean sleep bout time without impacting the mean wake bout time, and *vice versa* (independent control), while modifying excitation to either of the two populations necessarily impacts both sleep and wake mean bout times. Experimental evidence indicates that independent control of mean sleep and wake bout times is likely to be a physiological phenomenon, since alterations in the development of sleep bouts during early infancy tends to have little impact on the development of wake bouts, and *vice versa* (Karlsson et al., 2004; Karlsson et al., 2005; Gall et al., 2009). Our model therefore postulates that, in order to achieve independent control of sleep and wake bout times, the increase in mean sleep and wake bout lengths during P2-P10 may be due to modifications in the strength or temporal dynamics of cross-population synaptic inhibition between the sleep-active and wake-active populations.

The model further predicts that, in order to maintain independent control of sleep and wake bouts, there must be complete or near-complete silencing of the quiescent population during a bout. If inhibition from population *A* to population *B* is strengthened, then population *A*’s mean bout time increases, and population *B*’s mean bout time is unaffected because population *A* does not spike during a bout of population *B*. If population *A* were to maintain a small though significant level of spiking during a bout of population *B*, then the strengthened population *A* to population *B* inhibitory synapses would tend to decrease the duration of population *B*’s bouts, destroying independent control. Indeed, in accordance with this prediction of the model, empirical data show nearly complete silencing and lack of spiking in the sleep-active or wake-active population during a wake or sleep bout, respectively (Karlsson and Blumberg, 2005; Karlsson et al., 2005).

## 3 Development of the locus coeruleus during the P2-P21 period

There is a remarkable transformation in LC dynamics from the P10-P21 period, which coincides rather precisely with the transition of the wake bout distribution from exponential to power law. From P2-P10, LC cells display extensive dendrodendritic gap junction coupling and globally synchronized, high-amplitude membrane potential oscillations and spiking at a frequency of ∼0.3 Hz. From P10-P21, however, gap junctions are pruned, the amplitude of the global oscillation dampens, and frequency of the global oscillation rises to ∼3 Hz, with oscillations and synchrony vanishing at P21 and beyond (Coyle and Molliver, 1977; Groves and Wilson, 1980; Williams and Marshall, 1987; Christie et al., 1989; Christie and Jelinek, 1993; Travagli et al., 1995; Ishimatsu and Williams, 1996; Christie, 1997). Given strong empirical evidence showing that the LC is responsible for the shift of the wake bout distribution from exponential to power law, we constructed a biophysical model of 120 cells in the LC network (Patel and Joshi, 2015) in order to ask: can gap junction pruning explain the changes in the oscillatory properties of the LC observed in the P10-P21 epoch?

The main features of the model (in simulating the P2-P10 period) are extensive gap junction coupling among neurons and slow synaptic inhibition within the LC network. Within the LC, gap junction coupling is weak (a 100 mV depolarization of an LC cell leads to an ∼2 mV depolarization in a gap junction-coupled cell) and slow (a membrane potential change in an LC cell leads to an ∼10-fold slower response in a gap junction-coupled cell), indicating that gap junctions are likely located in distal dendrites and that the coupling is too slow to transmit spikes (Christie et al., 1989; Travagli et al., 1995; Christie, 1997). Gap junctions in our model are similarly weak, slow, and do not transmit action potentials. LC neurons are synaptically coupled to each other via slow *α*_2_ adrenergic receptors (which are present both pre- and post-synaptically and persist throughout the lifetime of the LC), which generate long ∼1–2 s membrane potential changes (Groves and Wilson, 1980; Egan et al., 1983; Ennis and Aston-Jones, 1986; Christie, 1997); these synapses are incorporated into our model as well. Each cell in our model also receives an independently constructed noisy excitatory drive in accordance with experimental measurements of external excitation to neonatal rat LC cells (Cherubini et al., 1988).

Within our model, we find that in the presence of both extensive gap junction coupling and synpatic inhibition (simulating the P2-P10 period), network cells indeed exhibit synchronized, high amplitude oscillations at ∼0.3 Hz (as measured through the power spectrum of the network’s calculated local field potential), and that both gap junctions and synaptic inhibition are required for this behavior. Without gap junctions, there is neither synchrony nor oscillations–synaptic inhibition is insufficent, in the presence of the robust excitatory drive, to generate oscillations or synchronize membrane potentials and spiking across cells. In the absence of synpatic inhibition, there is synchrony but no oscillatory behavior, as extensive direct electrical coupling tends to lead to a coordinated, network-wide burst of spikes each time a few cells spike in concert, but the next spike burst occurs after a random amount of time. Thus, we find that gap junctions are required to synchronize cells and synaptic inhibition is needed to ensure periodic behavior–after a network-wide spike burst facilitated by extensive electrical coupling, synaptic inhibition pervades the network and the length of time before a subsequent spike burst is determined by the temporal dynamics of inhibitory synapses (Patel and Joshi, 2015).

To simulate the P10-P21 period within our model, we progressively eliminate gap junctions, and we find that as the gap junction coupling probability between two neurons is systematically decreased from all-to-all to 0.1, the oscillation frequency of the network concordantly rises to about ∼3 Hz while the amplitude of the global network oscillation progressively declines, with complete elimination of gap junctions yielding little detectable oscillatory activity or synchrony (in accordance with empirical observations). As gap junctions are pruned within the model, reduced direct electrical coupling among cells causes each spike burst to be comprised of a successively smaller subset of network neurons, with weaker and less homogeneously distributed synaptic inhibition ensuing from the spike burst–cells which receive less inhibition following the spike burst can then initiate the next spike burst (composed of a subset of network cells in a similar position) after a shorter period of time, increasing the frequency of the network oscillation as well as reducing its amplitude (since each spike burst is comprised of a smaller subset of network cells which then distribute less inhibition to the network). In other words, as gap junctions are pruned, the network “breaks up” into a progressively greater number of oscillating, out-of-phase clusters of cells. Thus, our model suggests gap junction pruning from P10-P21 is sufficient to account for the physiological changes observed in LC behavior during this period (Patel and Joshi, 2015).

Pervasive gap junction coupling within the pre- and post-natal brain, and gap junction pruning during infancy and childhood, are prominent developmental features, and evidence suggests that electrical synapses play a critical role in neurogenesis, cell migration, synaptic plasticity, and stimulus tuning (e.g., orientation tuning in visual cortex), as well as in the develoment of both macroscopic neural architecture (e.g., cortical columns) and the underlying microscopic wiring of chemical synapses (Sutor and Hagerty, 2005; Bruzzone and Dermietzel, 2006; Niculescu and Lohmann, 2014; Cao et al., 2023). The LC in particular exhibits dense, widespread innervation of numerous brain areas, and gap junction coupling among LC neurons presumably allows for the coordinated release of noradrenaline at the LC’s various target sites. Early developmental regulation of gap junction coupling within the LC may play a role in its ability to modulate a broad array of behavioral functions, including the *CO*_2_ ventilatory response (de Carvalho et al., 2014), REM sleep development (Garcia-Rill et al., 2008), and electrical coupling between cortical networks (Roerig and Feller, 2000); indeed, deficits in electrical coupling within the LC are thought to underlie some of the behavorial and neurological impairments seen in connexin knockout mice (Hormuzdi et al., 2001; Frisch et al., 2005). While the relationship between changes in gap junction coupling within the LC and the development of power law wake bouts remains to be empirically elucidated, the precise temporal concordance of these two developmental events, and the requirement of an intact LC for the shift of wake bouts from exponential to power law (Gall et al., 2009), is strongly suggestive of a causal connection. The question then arises: what is the nature of the interaction of the LC with sleep-active and wake-active populations such that the changes in LC dynamics seen in the P10-P21 epoch result in the transformation of the wake bout distribution from exponential to power law?

## 4 Sleep-wake switching during the P10-P21 period

Unfortunately, there is little empirical data on the nature of the interaction of the LC with sleep-active and wake-active populations during the early postnatal period. In order to put forth a hypothesis about the nature of this interaction, we developed a coarse-grained three-population model of a sleep-active population, wake-active population and the LC (Patel and Rangan, 2017). While an exponentially distributed bout length is characterized by a constant hazard rate (the instantaneous probability of a bout switch) throughout a bout, a power law distribution has a heavier tail than an exponential distribution, and is hence characterized by a continuously *decreasing* hazard rate throughout the duration of a bout. A continuously decreasing hazard rate implies that the longer a bout endures, the more likely that it will endure even longer. Within the context of power law wake bouts this suggests a source of progressively mounting excitation to the wake-active population (and hence progressively mounting inhibition to the sleep-active population) during a wake bout, and we therefore postulated reciprocal excitatory connections between the wake-active population and the LC. However, we find within the model that, during a sleep bout, active excitation from the LC to the wake-active population can alter the exponential nature of the sleep bout distribution (since LC → wake-active excitation can progressively increase the hazard rate during a sleep bout); we therefore also postulated strong inhibitory connections from the sleep-active population to the LC, which ensure that the LC is inactive during a sleep bout (when the sleep population is active), and is released from inhibition during a wake bout (when the sleep population is suppressed) (Figure 1B).

Within the model, we found that, in order to change the exponential character of the wake bout distribution, there must exist slow (on the order 10s of seconds) time scales of excitation between the LC and wake-active population. If reciprocal excitation between the LC and wake-active population occurs only through fast synpases, then at the beginning of a wake bout the LC and wake-active populations rapidly equilibrate and attain steady-state mean activity levels (rapid relative to the time scale of typical wake bouts); a steady-state mean activity level for the wake-active population entails a constant hazard rate throughout the duration of the wake bout and hence an exponential bout distribution. Furthermore, we found that slow time scales in the interaction between the wake-active population and LC likely exist in the wake-active → LC direction, with LC → wake-active synapses remaining fast. The reason for this is that, in the case of slow LC → wake-active synapses, after the end of a wake bout–once the wake population is suppressed and a sleep bout begins–excitation from the LC to the wake-active population will linger for a considerable length of time despite suppression of the LC by the sleep-active population, since the slow character of this LC → wake-active excitation implies a prolonged time scale of decay. This lingering excitation to the wake-active population can result in a non-constant hazard rate during the sleep bout (slowly decaying excitation to the wake-active population during a sleep bout would imply a decreasing hazard rate), breaking the exponential nature of the sleep bout distribution. Thus, we included fast LC → wake-active excitation and slow wake-active → LC excitation within the model (Patel and Rangan, 2017).

To simulate the P2-P10 period within the model, we set the LC population to exhibit global synchronized oscillations and spiking at ∼0.3 Hz (3–4 s period), and progressively strengthened inhibition between the wake-active and sleep-active populations. We found exponential sleep and wake bout distributions with the sleep bout mean increasing from 19.6 to 38.2 s and the wake bout mean increasing from 3.6 to 9.1 s as the strength of inhibition between the two populations was increased (with independent control of sleep and wake bout times), similar to empirical observations (Kleitman and Engelmann, 1953; Lo et al., 2002; Halász et al., 2004; Karlsson et al., 2004; Lo et al., 2004; Blumberg et al., 2005; Karlsson et al., 2005; Gall et al., 2009). The LC population was set to exhibit globally coherent oscillations and synchronized spiking at ∼0.3 Hz throughout these simulations, in accordance with known physiology (Coyle and Molliver, 1977; Groves and Wilson, 1980; Williams and Marshall, 1987; Christie et al., 1989; Christie and Jelinek, 1993; Travagli et al., 1995; Ishimatsu and Williams, 1996; Christie, 1997). Why does feedback excitation between the LC and wake-active population not alter the exponential character of the wake bout distribution? Due to the LC being “locked” into an intrinsic oscillatory state of coordinated spike bursts followed by long periods of quiescience mediated by synchronized synpatic inhibition, excitation from the wake population has little impact on LC activity during periods of quiescence (due to the strength of the pervasive synaptic inhibition) and can do little more than perhaps mildly strengthen LC activity during a spike burst. Furthermore, the period of the LC oscillation (3–4 s), and hence the interval between successive spike bursts, is relatively long in comparison to typical wake bouts, implying that the LC tends to exhibit a spike burst zero or one times during most wake bouts; thus, since LC spike bursts are rapid and infrequent (causing only brief and rare alterations in the steady-state activity level of the wake-active population), there is no systematic drift in the mean activity level of the wake-active population during a wake bout, implying a constant hazard during a wake bout and hence an exponential wake bout distribution.

To simulate the P10-P21 period within the model, we continued to progressively strengthen inhibition between the sleep-active and wake-active populations (in order to increase mean bout times), while also progressively decreasing the amplitude of the LC oscillation and simultaneously increasing its frequency up to 3 Hz, gradually shifting the LC from its “locked” oscillatory regime at P10 towards a desynchronized, nonoscillatory, continuously active regime at P21, in accordance with experimental observations (Coyle and Molliver, 1977; Groves and Wilson, 1980; Williams and Marshall, 1987; Christie et al., 1989; Christie and Jelinek, 1993; Travagli et al., 1995; Ishimatsu and Williams, 1996; Christie, 1997). We found that, as model paramters were gradually changed from P10 to P21 values, sleep bouts remained exponentially distributed with the mean sleep bout time increasing from 38.2 to 69.8 s, while the wake bout distribution gradually transformed from exponential to power law with the mean wake bout time increasing from 9.1 to 23.5 s, in agreement with experiment (Kleitman and Engelmann, 1953; Karlsson et al., 2004; Blumberg et al., 2005; Karlsson et al., 2005; Gall et al., 2009). The increase in mean sleep bout time occurred as a consequence of the increasing strength of inhibition from the sleep-active to wake-active population, while the changes in the wake bout distribution were due to two factors: 1) the increasing strength of inhibition from the wake-active to the sleep-active population; 2) the impact of the LC on the wake-active population becoming more pronounced. Both factors likely contributed to the increase in the mean wake bout time, but the transformation of the qualitative nature of the wake bout distribution was due to the LC alone. How did the changing LC dynamics lead to the transformation of the wake bout distribution from exponential to power law? As the LC shifts from its “locked” oscillatory regime to its desynchronized continuously active regime, it becomes more sensitive to excitation from the wake-active population (during a wake bout) as well as being able to have a more pronounced impact on the wake-active population during a wake bout (since the LC network tends to fire more frequently during a wake bout as the LC oscillation frequency rises along with mean wake bout length increasing). Thus, a positive feedback loop develops between the LC and wake-active population–the firing rates of both populations gradually rise throughout the duration of a wake bout, and if the firing rate of the wake-active population gradually rises during a wake bout, then the hazard rate gradually decreases through the duration of the bout, leading to a more power law-like wake bout distribution. However, nearer P10, the LC is less responsive to excitation from the wake-active population and less potent at impacting the activity of the wake-active population, and so the positive feedback loop does not lead to an appreciable rise in the firing rate of the wake-active population unless a wake bout endures for a relatively long period of time; hence, only the “tail” of the wake bout distribution shifts noticeably. As model paramaters are varied from P10 to P21 values, and the positive feedback loop between the LC and wake-active population becomes more efficacious, an appreciably rising wake-active population firing rate can be observed for shorter wake bout lengths, transforming the wake bout distribution towards the “head” of the distribution. At P21, the firing rate of the wake-active population begins rising at the inception of a wake bout, resulting in a power law wake bout distribution. The model therefore predicts that, from P10 to P21, the transformation of the wake bout distribution from exponential to power law occurs in a “tail” to “head” fashion (Patel and Rangan, 2017).

To our knowledge, no other models have studied the shift of the wake bout distribution from exponential to power law during the P10-P21 epoch, though other models have investigated sleep-wake switching in the mature system, long after the emergence of power law-distributed wake bouts (McCarley and Hobson, 1975; Chu-Shore et al., 2010; Diniz Behn and Booth, 2010; Behn and Booth, 2011; Stephenson et al., 2013; Mosqueiro et al., 2014). These models include features such as REM/NREM switching during sleep bouts and external influences such as circadian rhythms and the hypothalamic hypocretin system [which are likely less important or absent during early infancy (Blumberg et al., 2014; Tham et al., 2017; Frank, 2020)], and rely less on the LC, since experimental evidence suggests that the LC may not have a large impact on maintaining the state of wakefulness in the mature system (Jones, 1991). Our model, in contrast, focuses specifically on the P10-P21 period, prior to the emergence of REM/NREM sleep and when system dynamics appear to be constituted simply by mutual inhibition between brainstem sleep-active and wake-active populations (Blumberg et al., 2014), and during which the LC plays a pivotal role in the dynamics of wake bouts. Furthermore, our model includes the dramatically changing physiological behavior of the LC during the P10-P21 period, and provides a plausible, experimentally testable mechanistic hypothesis of the interaction of the LC with sleep-active and wake-active populations and how this interaction leads to the observed transformation of the wake bout distribution from exponential to power law, explaining the failure of this transformation to occur when the LC is lesioned (Gall et al., 2009).

## 5 Discussion

A summary of our proposed infant sleep-wake circuit, including the LC, is shown in Figure 1. The green arrow (→) indicates excitatory connectivity while a red, blunt arrow (⊣) indicates an inhibitory influence. External excitatory drive on the sleep and wake neurons is shown as a wavy, blue arrow. During P2-P9, the LC is only weakly influential on the sleep-wake circuit and both sleep and wake bout distributions are exponential, albeit with means that increase with age, possibly driven by increasing the strength of mutual inhibition. After P10, associated with gap junction pruning in the LC, neurons within the LC become less synchronized and their influence on sustaining an ongoing wake bout becomes more prominent. Due to this effect, between P10 and P21, the wake bout distribution develops a heavy tail without impacting the qualitative nature of the sleep bout distribution. Throughout development, both in experimental data and model behavior, we find independent control of bout durations–sleep and wake bout means increase independently of each other. Independent control is strongly suggested by experimental lesions of sleep-active populations leaving wake bouts unaffected and *vice versa*. A prediction of our model is that an increase in the strength of reciprocal inhibition, rather than an increase in the strength of the external excitatory drive, leads to independent control of sleep and wake bout durations.

An explicit goal of our modeling is hypothesis discrimination, and hence a possibility for future research is identifying other network mechanisms which lead to the same observed sleep-wake distributions. Sustaining a power law wake bout distribution over its entire duration requires a slow and sustained increase in the firing rate of the wake-active population over a wake bout. In our model, this occurs via a slower time scale excitation from the wake-active population to the LC. Experimental studies are needed to determine if such time scales exist, and to identify the precise synaptic mechanisms for the interplay of multiple time scales in the sleep-wake system, though until such empirical work is available, future modeling work can explore the dynamical feasibility of slower time scales of excitation introduced into various components of the sleep-wake circuit. While we explicitly modeled the change in the intrapopulation connectivity of the LC, interpopulation connectivity between the LC and sleep-active and wake-active populations remained fixed in our model through development; empirically, it is unknown whether the synaptic connectivity of the LC with sleep-active and wake-active populations changes over time and whether this plays a role in sleep regulation, a feature which could be explored in future modeling work. Sleep plays an important role in brain development (Blumberg et al., 2020; Del Rio-Bermudez et al., 2020; Frank, 2020), and a long-term challenge is to integrate the infant sleep-wake model into a model of long-term development into adulthood, which would require progressive incorporation of the processes that naturally lead to the age-related transition from the infant to the adult sleep-wake circuit, including features such as circadian rhythms, cortical and hypothalamic influences on the brainstem sleep-wake circuit, and subdivisions of sleep into REM/NREM phases and sleep stages.

The functional and behavioral significance of the early developmental changes in sleep and wake bouts remains unclear and the subject of controversy. Sleep consolidation–i.e., increases in mean bout length and the emergence of subdivisions of sleep–over the developmental period has been proposed to be important in numerous areas, including normal synaptic development and brain maturation (with REM and non-REM sleep states possibly playing distinct but complementary roles) (Peirano and Algarin, 2007), synaptic plasicity, learning, and the consolidation of declarative as well as procedural memories (Walker and Stickgold, 2004), cognitive development (Mason et al., 2021), and macroscopic and microscopic structural brain development (Lokhandwala and Spencer, 2022). While this remains an area of active investigation, precise physiological mechanisms and functional implications remain, in general, to be elucidated. However, while the developmental transition of wake bouts from exponential to power law is observed across species (Lo et al., 2004), the functional and behavioral significance of this qualitative shift in the wake bout distribution remains entirely speculative. If the shift to power law wake bouts reflects an underlying development of a scale-free network structure in wake-active populations, then the shift could confer resistance to damage to wake-active populations, since scale-free networks are robust to random (non-targeted) attacks (and are vulnerable only to targeted attacks on hubs) (Gall et al., 2009). Behaviorally, power law wake bouts–in which the likelihood of a switch to sleep decreases with wake bout length–could be a mechanism for preventing the termination of prolonged wake bouts (i.e., preventing sleep from intruding inappropriately into the waking period, which could be harmful). Unfortunately, there is little evidence to either support or refute any such speculations, and hence this mystery awaits further empirical data.

## Data Availability

The original contributions presented in the study are included in the article/supplementary material, further inquiries can be directed to the corresponding author.
